# First Report of Imported Fire Ants, *Solenopsis invicta, S. richteri,* and *S. invicta* X *richteri* (Hymenoptera: Formicidae) from Kentucky

**DOI:** 10.3390/insects14040372

**Published:** 2023-04-10

**Authors:** Jennifer L. Seltzer, Joe MacGown, JoVonn G. Hill, David Cross, Janet Lensing, Joe Collins

**Affiliations:** 1 Mississippi Entomological Museum, Department of Biochemistry, Molecular Biology, Entomology & Plant Pathology, Mississippi State University, Starkville, MS 39762, USA; 2 Department of Biochemistry, Molecular Biology, Entomology & Plant Pathology, Mississippi State University, Starkville, MS 39762, USA; 3 Kentucky’s Office of the State Entomologist, University of Kentucky, Lexington, KY 40546, USA

**Keywords:** invasive species, insect identification, range expansion

## Abstract

**Simple Summary:**

Imported fire ants, an invasive species, are reported from multiple locales in Kentucky, USA from 2014 to 2022.

**Abstract:**

Since their introduction into the United States in the early 1900′s, imported fire ants, namely *Solenopsis invicta* Buren (Red Imported Fire Ant), *S. richteri* Forel (Black Imported Fire Ant), and their hybrid form *Solenopsis invicta* X *richteri* have spread throughout portions of the USA, especially in the southeastern region. Imported fire ants are a serious invasive and economically significant species in the USA and elsewhere, and their spread into new parts of the country is of great concern. Although early models predicted that the fire ants would not be able to survive very far north into the USA, these ants have nonetheless successfully continued their spread into higher latitudes. Based on Cooperative Agricultural Pest Survey (CAPS) samples, the Mississippi Entomological Museum Invasive Insect Screening Center, at Mississippi State University, has verified the presence of imported fire ants collected in Kentucky at multiple locations from 2014 to 2022.

## 1. Introduction

The Mississippi Entomological Museum Invasive Insect Screening Center, funded by the USDA Plant Protection Act Section 7721 (PPA7721), was established by the Mississippi Entomological Museum (MEM) at Mississippi State University to provide taxonomic services for Federal and state invasive insect survey programs in the eastern USA. These services were designed to speed up the identification and early detection of exotic species. Samples are received via in-person drop-off and mail services from participating state Cooperative Agricultural Pest Survey (CAPS) programs, PPA7721 Goal 1 Survey participants, and USDA Plant Protection and Quarantine programs in the USA. Upon arrival, samples are screened for target taxa and possible exotics are then examined by experts. Positive identifications are reported, and voucher specimens are pinned, labeled, and deposited in the Mississippi Entomological Museum (MEM) with data entered into the Symbiota Collections of Arthropods Network (SCAN). Additionally, representatives of native target taxa are also retained and curated into the museum collection. The deposition of accurately labeled and identified specimens into museum collections and reporting of findings are critical components of the monitoring process. 

Imported fire ants (Hymenoptera: Formicidae) including *Solenopsis invicta* Buren (Red Imported Fire Ant), *Solenopsis richteri* Forel (Black Imported Fire Ant), and their hybrid form *Solenopsis invicta* X *S. richteri,* are native to South America and are seriously invasive and economically significant species in the USA and elsewhere. Here we report records of *S. invicta, S. richteri,* and *S. invicta* X *S*. *richteri* from Kentucky, provide information about identification, discuss their economic and medical importance, and give a brief history of their spread in the region. 

### 1.1. Identification

Imported fire ants can easily be detected in the field by the large mounds they typically construct, their aggressive stinging behavior, relatively small size (workers are 2.4–6.0 mm in total length), and black and red coloration. Other diagnostic characteristics for the worker caste include the antennae having 10 antennomeres of which the apical two are enlarged forming a club, propodeum lacking adornment in the form of spines or other protuberances, waist with two nodes, and gaster with a prominent sting. Females are similar to workers, but larger (7.0–7.5 mm in overall length) and have 11 antennomeres. Males are slightly larger than large workers (5.0–6.0 mm in total length), body concolorous black, head tiny in proportion to the body, eyes enlarged, and have 12 antennomeres [1]. 

**Figure 1:**
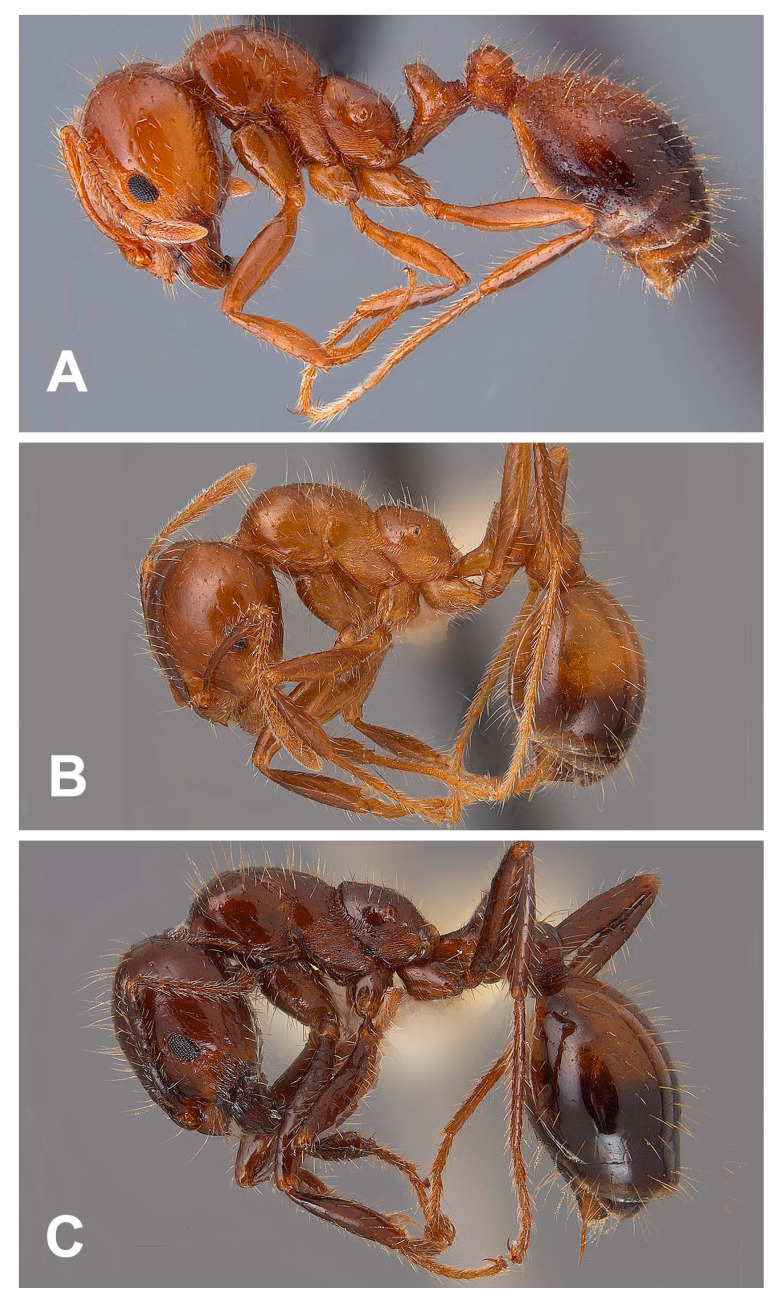
Lateral habitus views of imported fire ant workers: (**A**) *Solenopsis invicta*, (**B**) *S. invicta* X *richteri*, and (**C**) *S. richteri*.

Distinguishing *S. invicta* from *S. richteri* may be difficult where their geographic ranges overlap because they create hybrids that have a mixture of features from the two species. Definitive identifications may require examination of cuticular hydrocarbons and venom alkaloids or DNA analysis [2,3]. In general, *S. richteri* (Figure 1C) can be separated from *S. invicta* (Figure 1A) by its overall darker coloration, dark brownish-black antennal scapes, and more prominent pronotal humeri. Workers of the hybrid form (Figure 1B) vary from mostly having characteristics of *S. invicta* to being very similar to *S. richteri*, with variation between the two being common, depending on the amount of DNA present in either species in their genetic makeup. It is not unusual to find considerable variation in workers within a single colony of the hybrid form. 

Nests are typically in the soil and colonies are usually surmounted by large mounds up to one meter wide and tall. Mature colonies may be large with 500,000 workers or more, and in the case of *S. invicta* and the hybrid, colonies may be polygyne. Nests are usually in open areas such as agricultural areas, rights of way, pastures, grasslands, and urban landscapes with mounds numbering up to 300 per acre if conditions are suitable [4,5]. 

### 1.2. Economic Impact

The USDA estimates annual losses resulting from damage and control of imported fire ants in the USA to be over five billion dollars [5]. Negative effects of these invasive ants in natural systems include predation of nesting songbirds [6], ground-nesting birds, and other wildlife [5]. They impact agriculture in numerous ways such as by damaging dry seeds [7], feeding on buds of crop plants, girdling young trees, attacking young livestock, damage to farm equipment from large mounds, damaging turf and lawns, and more. Similar to other ants, fire ants may cause disruption to electrical systems [5]. Perhaps most importantly from a human perspective, reactions from the stings of fire ants range from minor pain to death in a small number of cases. 

### 1.3. Medical Importance 

Imported fire ants aggressively attack and sting intruders including humans, especially when colonies are disturbed. Oi [8] estimated that 30–60% of the people living in areas of the USA infested by imported fire ants are stung each year. Reactions to stings in most people include intense burning, itching, redness, and a small, raised welt that in many individuals results in a pustule with a white blister at the top. Anaphylactic reactions include systematic itching, welts, weakness, difficulty in swallowing and/or breathing, fainting, and even death in a small percentage of the population [9,10]. It is difficult to know exactly how many individuals experience anaphylactic reactions from fire ant stings in the USA because reports vary widely from 0.6 to 16 percent [9]. A review by deShazo et al. [9] reported 80+ cases of deaths in the USA resulting from anaphylactic reactions from stings. As fire ants expand their range, these numbers will likely increase. 

### 1.4. How Fire Ants Spread 

Imported fire ants spread geographically both from natural mating and dispersal methods and by anthropogenic movement. Fire ants may disperse naturally by localized relocation of colonies due to disturbance. Recently mated queens have been documented to fly up to 1.6 km over land and up to 16 km over open water (probably wind-aided) to new areas to start colonies [11], and queens may be blown by the wind for even further distances [5]. Colonies flooded by water can form living mats of ants that can survive up to several weeks on the surfaces of ponds, lakes, and rivers. If they drift ashore or the flooded areas recede, the ants can reestablish colonies on dry land, sometimes miles away [11]. 

Human activities play a significant role in the spread of invasive species including imported fire ants. Newly mated queens may be transported on motor vehicles on which they have landed. Colonies may be transported in the soil of nursery stock, mulch, sand, gravel, grass, sod, hay, wood, beehives, soil-moving equipment, and other inadvertent means [5,11]. 

### 1.5. History of Imported Fire Ant Spread in the USA

Both *S. richteri* and *S. invicta* are thought to have first arrived via cargo ships in the USA in Mobile, Alabama with *S. richteri* arriving as early as 1918 and *S. invicta* likely in the early 1930s [11]. It should be noted that *S. invicta* and *S. richteri* were not formally recognized as two distinct species until 1972, when Buren revised the group [12], although two distinct color morphs were known to be present. Historical communications by Loding to Creighton [13] indicated that the first fire ants observed in the Mobile area were of a dark form, with the red form arriving later. Trager [14], after examining historical specimens from Mobile, stated that he believed hybridization had occurred between the two species. These two closely related species have a large area of range overlap in their native range, and yet hybridization occurs there only rarely. When *S. invicta* was introduced to the USA, it proved to be the more aggressive species and apparently displaced *S. richteri* from the Mobile area, with the idea being that *S. richteri* was continuously being forced east, north, and west by *S. invicta.* Trager [14] wrote that *S. richteri* “apparently occupied much of Mississippi and Alabama”, but specimen records do not reflect this. Instead, the movement of *S. richteri* may have been less linear, and instead patchier due to incidental movement by trains or other means. Historical records indicate that *S. richteri* (and possibly a hybrid form) was forced outward from the Mobile area into the southeastern coastal area of Mississippi and southwestern Alabama. By 1949, *S. richteri* was no longer found in those areas and was replaced by populations of *S. invicta* [11]. However, before they were eliminated from the Mobile region, noncontiguous populations appeared in Artesia, MS as early as 1935 [10], Meridian, MS as early as 1940 [11], Sessums, Mississippi (1952, MEM Records) and Selma, Alabama as early as 1944 [11]. These seemingly disconnected towns in central Mississippi and Alabama all shared railroad connections, and it is likely that *S. richteri* populations in those localities resulted from accidental introductions via trains. Although *S. richteri* was eliminated from the coast by fierce competition of *S. invicta,* conditions and isolation from *S. invicta* in these locations in central Alabama and Mississippi appeared to have allowed *S. richteri* to gain a stronger foothold. Eventually, *S. invicta* continued its spread and when it reached these northern populations of *S. richteri*, hybridization occurred between the two species at the edges of their distributions and pure populations of *S. richteri* were again pushed northward. This northward spread of *S. richteri,* stemming primarily from the disjunct central Mississippi populations, has continued north through western Tennessee and most recently into southwestern Kentucky, with *S. invicta* surrounding populations of *S. richteri* both to the south, west, and the east and the hybrid form forming a distinct “ring” between the two species. *Solenopsis richteri* has been reported in the literature from Arkansas [15,16], but these records were from specimens collected before *S. richteri* was formerly recognized as a species and these unverified records seem unlikely. Examination of historical museum specimens from Arkansas by MacGown, as well as numerous collections by MEM staff and other researchers in Arkansas corroborate the idea that *S. richteri* has not been found in Arkansas. 

Based on habitat and climate in their native ranges, a model of range expansion in the USA given by Korzukhin et al. [17] predicted that the northward spread of *S. invicta* would be limited to a latitude of approximately 42° along the Pacific coastline and approximately 37° elsewhere based on the cold tolerance of the ants. The authors did not estimate cold tolerance for *S. richteri* or *S. invicta* X *richteri* but speculated that they may have slightly more tolerance for cold temperatures than *S. invicta.* Overall, the model given by Korzukhin et al. [17] has proven to be fairly accurate in predicting that *S. invicta* would spread westward to California to the southern Oregon border and eastward up the Atlantic coast as far north as Virginia. However, imported fire ants have spread further north than predicted and have been found in areas once thought to have been improbable such as Oregon, Washington D.C., Maryland, and most recently Kentucky. 

## 2. Materials and Methods

Specimens from possible fire ant mounds reported by Kentucky citizens were collected by CAPS employees and placed in ethanol and/or hexane and sent to the MEM Invasive Insect Screening Center for identification and verification. Identifications were made using morphological methods and the keys found in [1]. When possible, identifications were verified using DNA analysis based on methods provided by Goodisman et al. [3] and using gas chromatography and mass spectrometer (GC/MS) methods to separate *S. invicta*, *S. richteri*, and their hybrid from one another based on different cuticular hydrocarbon and venom alkaloid profiles as described in Menzel et al. [2]. Specimens collected in ethanol were adequate for both DNA and morphological-based identifications, whereas samples stored in hexane were necessary for the GC/MS analyses. Photomicrographs were captured using a Leica DFC 495 digital camera mounted on a Leica Z16 Microscope with motorized Z-stepping, and image stacks were merged using Leica Application Suite V 4.1.0 with the Montage Module. All images were edited in Photoshop CS6. Voucher specimens were deposited in the MEM. 

## 3. Results

**Figure 2:**
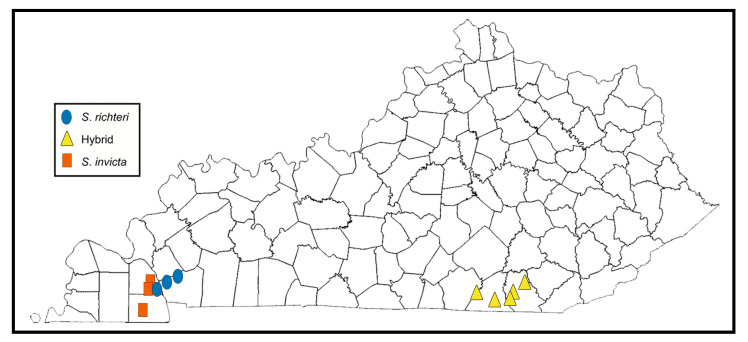
Map of Kentucky showing counties where imported fire ants have been reported.

Though the model given by Korzukhin et al. [17] predicted that imported fire ants were unlikely to be found in Kentucky due to its cooler climate, colonies were detected as early as 2009 in Calloway County via CAPS surveys [15]. Since that point in time, populations have been collected in four more counties to date (Figure 2). The presence of fire ants in Kentucky and other northern localities is likely a result of climate change and the possible adaption of the ants to colder temperatures. 

The earliest observations of imported fire ants in Kentucky were in October 2009 when mounds were detected during a CAPS survey at a nursery in Murray in Calloway County in the southwestern portion of the state [18]. Mounds were treated with insecticide and representative specimens were collected and determined to be *S. invicta*. These specimens were not corroborated by the MEM. Three years later in 2012, fire ant mounds were again detected in Calloway County and confirmed by a USDA identifier as *S. invicta* [18]. 

In July 2013, United States Forest Service personnel found fire ant mounds in the Land between the Lakes National Recreational Area in Trigg County, which is located in the southwestern part of the state. Specimens were collected and identified as *S. richteri* by a USDA identifier. The following year, a sample collected in Trigg County at the Lake Barkley Marina (36.8530–87.9422) on 14 October 2014 by J. Lensing was sent to the MEM for verification and specific identification. Specimens, which had been collected in ethanol, were identified as *S. richteri* based on morphological characteristics, and genetic assays agreed with this determination. In 2015, the MEM received additional samples of fire ants from Trigg County collected near the Golden Pond Visitor Center (36.7797–88.0670) on 19 May 2015 by J. Lensing. Samples were again collected in ethanol and based on morphological examinations and genetic assays the ants were determined to be *S. richteri*. Another sample was made in Trigg County near the Golden Pond Visitor Center (36.7797–88.0670) on 1 August 2015 by J. Lensing and sent to the MEM. Specimens were collected in both ethanol and hexane and again determined to be *S. richteri*. The hexane sample was very helpful as it allowed us to corroborate the *S. richteri* using gas chromatography and mass spectrometry methods. 

In 2017, 79 fire ant mounds were detected at the Kenlake State Resort Park in Marshall County, and specimens were identified as *S. invicta* [18]. On 18 September 2017, fire ants were collected in two nearby sites (36.7677–88.1327 and 36.7673–88.1323) near the Kenlake Tennis Center at the Kenlake State Resort Park by J. Lensing. Samples were sent to the MEM Screening Center for determination and based on GC/MS, genetic assay, and morphology, the specimens were identified as *S. invicta*, which corroborated earlier identifications of fire ants from the same site. All mounds at the Kenlake sites were treated beginning in September 2017 and retreated in October 2017. The treatments appeared to be successful as no fire ants were found on return visits to the site after the collections were made in September 2017. Likewise, fire ants were not detected there during surveys in 2019 and 2021 [18]. Although the fire ants appeared to have been successfully eradicated from the Kenlake sites, mounds were again found, and subsequently treated with insecticide, near the Kenlake Tennis Center on 15 March 2022 by J. Collins. However, based on a morphological examination, specimens were determined to be *S. richteri*, rather than *S. invicta*, which had been previously found at the site in 2017 and 2018. 

In 2022, the MEM received samples of fire ants collected by J. Collins from McCreary and Whitley Counties in the southeastern part of the state. Data for specimens were as follows: KY, McCreary Co., Strunk, 36.6409, −84.4100, 21 February 2022, J. Collins, collected at farm/residence; KY, McCreary Co., Stearns, 36.7164, −84.6099, 14 June 2022, J. Collins, collected at residence; KY, Whitley Co., Williamsburg, 36.6853, −84.2395, 22 September 2022, J. Collins, collected on roadside; KY, Whitley Co., Williamsburg, 36.7732, −84.0765, 22 September 2022, J. Collins, collected on farm; KY, Whitley Co., Williamsburg, 36.6409, −84.2589, 21 June 2022, R. Bessin, collected on farm. All samples from McCreary and Whitley Counties were morphologically identified as *S. invicta* X *richteri*.

## 4. Discussion

Based on results from a recent paper by Pandey et al. [19] on the distribution and hybridization of *S. invicta* and *S. richteri* in Tennessee, *S. richteri* populations were observed only in the western portion of the state from the northern border of Mississippi to near the southwestern border of Kentucky. According to Pandey et al. [19], populations of *S. invicta* only comprised 2.3% of imported fire ants found during a survey done in Tennessee from 2004–2005, and colonies were only found in a few sites in the central and eastern parts of the state, mostly in metropolitan areas. Populations of the hybrid form were found to be widespread across the entire southern half of the state [19]. 

Thus far, populations of *S. richteri* collected in Kentucky have only been found in the southwestern region directly north of a line following the distribution of this species from Mississippi through west and central Tennessee [19]. Similarly, localities where the hybrid form has been recently found in eastern Kentucky are directly north of the region in eastern Tennessee where hybrid fire ants have become widespread [19]. 

Given that *S. invicta* is thought to be less cold tolerant than *S. richteri* or their hybrid form [17], its presence in Kentucky was unanticipated. The closest populations of *S. invicta* reported from Tennessee were from Nashville, and that was the only locality in the entire western two-thirds of the state where it has been observed. It would not be a stretch to think that fertilized females of *S. invicta* might have been inadvertently transported from the Nashville area via truck along Interstate Highway 24 and Highway 80 to the site in Murray, Kentucky where this species was first found at a plant nursery. Due to *S. invicta* being less cold tolerant, it is probable that eradication efforts were successful. The discovery of *S. invicta* several years later in 2017 at the Kenlake State Resort Park was also likely due to anthropogenic movement of the species, as the area is known for outdoor recreational activities and fire ants could have easily been transported to the region. At first glance, it may appear surprising that both *S. invicta* and *S. richteri* were found in the same area at Kenlake Park, however, their occurrences there did not overlap temporally. Early populations of *S. invicta* were apparently eradicated, allowing *S. richteri* to successfully colonize the area later. 

Imported fire ants are clearly moving farther north than predicted, both naturally and by unintentional anthropogenic transport. As a result of the possible adaptation of the ants to colder temperatures and our climate becoming slightly warmer, imported fire ants are now likely established in Kentucky. Diligence when transporting various goods from quarantined areas is essential. Reporting to the Kentucky CAPS program of imported fire ant mounds is also critical in stemming their spread. 

## Data Availability

Specimen data from this project has been uploaded to the Symbiota Collection of Arthropod Networks (SCAN), https://scan-bugs.org/portal/index.php (accessed on 21 February 2023).
